# Harmony in Chaos: Deciphering the Influence of Ischemic Cardiomyopathy and Non-Cardiac Comorbidities on Holter ECG Parameters in Chronic Heart Failure Patients: A Pilot Study

**DOI:** 10.3390/medicina60020342

**Published:** 2024-02-19

**Authors:** Ștefania-Teodora Duca, Minerva Codruta Badescu, Alexandru-Dan Costache, Adriana Chetran, Radu Ștefan Miftode, Ionuț Tudorancea, Ovidiu Mitu, Irina Afrăsânie, Radu-George Ciorap, Ionela-Lăcrămioara Șerban, D. Robert Pavăl, Bianca Dmour, Maria-Ruxandra Cepoi, Irina-Iuliana Costache-Enache

**Affiliations:** 1 Department of Internal Medicine I, Faculty of Medicine, University of Medicine and Pharmacy “Grigore T. Popa”, 700115 Iasi, Romania; stefania-teodora.duca@email.umfiasi.ro (Ș.-T.D.); adriana.ion@umfiasi.ro (A.C.); radu-stefan.miftode@umfiasi.ro (R.Ș.M.); ovidiu.mitu@umfiasi.ro (O.M.); irina.afrasanie@umfiasi.ro (I.A.); bianca-ana-dmour@email.umfiasi.ro (B.D.); cepoi_maria-ruxandra@d.umfiasi.ro (M.-R.C.); irina.costache@umfiasi.ro (I.-I.C.-E.); 2 Department of Cardiology, “St. Spiridon” Emergency County Hospital, 700111 Iasi, Romania; ionut.tudorancea@umfiasi.ro; 3 Department of III Internal Medicine Clinic, “St. Spiridon” Emergency County Hospital, 700111 Iasi, Romania; 4 Department of Cardiovascular Rehabilitation, Clinical Rehabilitation Hospital, 700661 Iasi, Romania; 5 Department of Morpho-Functional Science II-Physiology, University of Medicine and Pharmacy “Grigore T. Popa”, 700115 Iasi, Romania; ionela.serban@umfiasi.ro; 6 Department of Biomedical Science, Faculty of Medical Bioengineering, University of Medicine and Pharmacy “Grigore T. Popa”, 700145 Iasi, Romania; radu.ciorap@umfiasi.ro; 7 Faculty of Health Sciences and Sport, University of Stirling, Stirling FK9 4LA, UK; d.r.paval@stir.ac.uk

**Keywords:** ischemic cardiomyopathy, Holter ECG, comorbidities, chronic heart failure

## Abstract

*Background and Objective*: In the landscape of heart failure, non-cardiac comorbidities represent a formidable challenge, imparting adverse prognostic implications. Holter ECG monitoring assumes a supplementary role in delineating myocardial susceptibility and autonomic nervous system dynamics. This study aims to explore the potential correlation between Holter ECG parameters and comorbidities in individuals with ischemic cardiomyopathy experiencing heart failure (HF), with a particular focus on the primary utility of these parameters as prognostic indicators. *Materials and Methods*: In this prospective inquiry, a cohort of 60 individuals diagnosed with heart failure underwent stratification into subgroups based on the presence of comorbidities, including diabetes, chronic kidney disease, obesity, or hyperuricemia. Upon admission, a thorough evaluation of all participants encompassed echocardiography, laboratory panel analysis, and 24 h Holter monitoring. *Results*: Significant associations were uncovered between diabetes and unconventional physiological indicators, specifically the Triangular index (*p* = 0.035) and deceleration capacity (*p* = 0.002). Pertaining to creatinine clearance, notable correlations surfaced with RMSSD (*p* = 0.026), PNN50 (*p* = 0.013), and high-frequency power (*p* = 0.026). An examination of uric acid levels and distinctive Holter ECG patterns unveiled statistical significance, particularly regarding the deceleration capacity (*p* = 0.045). Nevertheless, in the evaluation of the Body Mass Index, no statistically significant findings emerged concerning Holter ECG parameters. *Conclusions*: The identified statistical correlations between non-cardiac comorbidities and patterns elucidated in Holter ECG recordings underscore the heightened diagnostic utility of this investigative modality in the comprehensive evaluation of individuals grappling with HF. Furthermore, we underscore the critical importance of the thorough analysis of Holter ECG recordings, particularly with regard to subtle and emerging parameters that may be overlooked or insufficiently acknowledged.

## 1. Introduction

Heart failure (HF) is an intricate clinical syndrome that arises from anomalies, be they structural or functional, within the myocardium. This results in elevated intracardiac pressures and/or a reduction in the cardiac output [1]. The imperative task of accurately diagnosing and tailoring treatment demands the identification of the underlying etiology responsible for cardiac dysfunction [2]. The classification of heart failure is no easy feat, given its inherent heterogeneity. The current stratification based on the left ventricular ejection fraction (LVEF) split into subtypes—heart failure with a reduced ejection fraction (HFrEF), heart failure with a mildly reduced ejection fraction (HFmrEF), and heart failure with a preserved ejection fraction (HFpEF)—offers a glimpse into this complexity [3]. A novel classification, HF with improved EF, factors into temporal changes in the LVEF [4,5,6].

In tandem with the New York Heart Association (NYHA), functional classification, which assesses heart failure severity based on symptoms, alternative prognostic markers such as N-terminal pro-B-type natriuretic peptides (NT-proBNP), becomes pivotal. This is especially true considering the heightened risk for hospitalization and mortality, even in individuals with mild symptoms [1,3]. The extensively researched NT-proBNP serves as a prognostic biomarker, providing diagnostic and prognostic insights into HF with a reduced ejection fraction, and its predictive capacity extends to the broader population [7,8,9,10,11].

While imaging techniques unravel structural changes indicative of the myocardial substrate, Holter monitoring plays a complementary role in offering insights into both myocardial vulnerability and autonomic nervous system dynamics. Recent interest focuses on dynamic Holter-derived ECG markers, such as ventricular late potentials (VLPs), discerned through a signal-average electrocardiogram. VLPs hold implications for predicting sudden cardiac death and lethal arrhythmias, particularly in the context of organic heart diseases [12,13,14,15]. The combined assessment of VLPs with T-wave alternans (TWA) and heart rate variability (HRV) provides a more comprehensive evaluation [15,16,17,18].

HRV analysis, a non-invasive method for assessing autonomic function, proves valuable in predicting cardiovascular death across diverse clinical populations [19,20,21]. However, the nuanced nature of HRV indexes is often underappreciated, leading to potential misconceptions. In 2006, Baver et al. introduced a methodological advancement to differentiate vagal and sympathetic nervous system roles [21,22,23]. A reduced heart rate deceleration capacity emerged as a robust predictor of mortality, independent of LVEF [24,25,26,27,28,29].

T-wave alternans (TWA), a prevalent manifestation of cardiac electrical alternans characterized by repolarization dispersion, acts as a biomarker in predicting malignant arrhythmias and sudden cardiac death [30,31,32,33]. Substantial empirical evidence underscores its efficacy in predicting outcomes, demonstrating clinical relevance across diverse patient cohorts, including those with heart failure and ischemic cardiomyopathy [33,34,35].

Comorbidities, notably diabetes, hyperuricemia, chronic kidney disease (CKD), and obesity, present a significant challenge in HF, contributing to adverse prognostic implications [36,37]. This research exclusively delves into these non-cardiovascular comorbidities, scrutinizing their role in the emergence of T-wave alternans, VLPs, and alterations in the HRV and the acceleration and deceleration capacity, influenced by various underlying mechanisms [30,38,39,40].

## 2. Materials and Methods

### 2.1. Study Design and Investigations

We conducted a prospective investigation involving 60 consecutively enrolled patients presenting with heart failure (HF) and left ventricular ejection fraction (LVEF) of less than 50%. The study was conducted at St. Spiridon County Hospital in Iasi, Romania, spanning from May 2023 to October 2023. Throughout their hospitalization in the Cardiology Department, we diligently monitored the participants’ conditions. All study subjects had a pre-existing diagnosis of systolic heart failure with LVEF of less than 50%. To adhere to predetermined eligibility criteria and to prevent instances of acute heart failure or acute decompensation of chronic heart failure, participants were required to demonstrate clinical stability for a minimum of one month before the collection of biomarker samples and the initiation of Holter ECG/24 h monitoring.

Importantly, candidates had to exhibit an absence of rapid or gradual onset of symptoms and signs indicative of heart failure of sufficient severity to necessitate the initiation or escalation of treatment, including intravenous therapies. Additionally, participants were mandated to display an absence of indications corresponding to the principal clinical presentations, specifically, acute decompensated heart failure, acute pulmonary edema, isolated right ventricular failure, or cardiogenic shock.

Inclusion of participants in the study was contingent upon a verified etiology of ischemic heart failure. The classification of ischemic heart failure was based on the concurrent manifestation of clinical heart failure and the confirmation, through historical medical records, of atherosclerotic coronary lesions surpassing 75%, as determined by coronary arteriography. Each subject underwent coronary angiography at least one month prior to inclusion, revealing a minimum of 75% atherosclerotic coronary lesions in at least one coronary artery. Consequently, participants exhibited a pathological spectrum inclusive of unicoronary, bicoronary, or tricoronary lesions at the point of enrollment.

Exclusion criteria involved patients who declined to provide informed consent upon admission and those unable to undergo a comprehensive physical and/or echocardiographic examination due to factors like recent thoracic surgery, severe thoracic malformations, or heightened sensory perception (hyperesthesia). Patients with active malignancies or those under antineoplastic medication, individuals with comorbid conditions associated with a life expectancy of less than one year, and patients taking potentially arrhythmogenic medications were also excluded. Additionally, patients with concurrent acute or chronic inflammatory processes, thyroid disorders, recent major surgical procedures, untreated neuropsychiatric disorders, or NT-proBNP values upon admission falling below the recommended cutoff of 125 pg/mL by the European Society of Cardiology were excluded. Furthermore, specific patient groups with attributes that could potentially confound the interpretation of study outcomes were excluded. This criterion encompassed individuals with a recent history of acute coronary syndrome within 21 days prior to admission, those with a documented history of sustained ventricular tachycardia or sudden cardiac death, patients in atrial fibrillation, and individuals with cardiac pacemakers. The study’s scope did not include individuals under the age of 18 or pregnant women.

After obtaining informed consent, patients underwent an extensive clinical assessment, standard laboratory investigations, and a series of noninvasive diagnostic procedures. These noninvasive diagnostic methods included an ECG, 24 h Holter monitoring, signal-averaged electrocardiography (SAECG), and a transthoracic echocardiographic examination.

Our comprehensive patient assessment involved a detailed examination of medical history, a standard physical examination, and a thorough review of patients’ medical records or archival data from the hospital. This review encompassed the extraction of relevant sociodemographic information, specific behavioral conditions, underlying medical conditions, current medication regimens, clinical observations, and laboratory findings. To ensure scientific rigor and reproducibility in our statistical analysis, we opted to utilize data collected exclusively at the time of patient enrollment, considering the potential variability in biomarker concentrations and clinical parameters such as blood pressure, weight, height, and body mass index over time. The confirmation of comorbidities was based on either pre-existing records in the patients’ personal files or diagnoses made during their hospitalization, adhering to established diagnostic criteria.

We systematically conducted an extensive array of standardized laboratory tests covering diverse parameters, including NT-proBNP, D-dimers, complete blood count, renal and hepatic function, C-reactive protein, sodium, potassium, magnesium, microalbuminuria, uric acid, total protein test, thyroid function markers, serum iron, ferritin, HbA1c, and glycemia. The primary objective of this thorough battery of laboratory tests was to rule out potential underlying causes, such as infection, electrolyte imbalances, hepatic dysfunction, hypoalbuminemia, anemia, or thyroid disorders. NT-proBNP levels were quantified using the PATHFAST Cardiac Biomarker Analyzer (LSI Medience Corporation, Tokyo, Japan), employing a chemiluminescent enzyme immunoassay and the MAGTRATION^®^ method. The manufacturer’s designated reference range for NT-proBNP fell within <15–128 pg/mL.

Initiating a thorough evaluation, each patient underwent a detailed echocardiography assessment utilizing the GE VividTM V7 ultrasound system (General Electric, Boston, CA, USA). The determination of LVEF followed the standardized protocol, employing Simpson’s method within the two-dimensional echocardiographic apical four-chamber view.

Patients underwent 24 h Holter ECG monitoring utilizing a twelve-channel CardioScan DMS 300–3L, a digital recorder with ten wires manufactured by DM System Company Ltd., Beijing, China. Subsequently, all recorded data underwent meticulous manual scrutiny, with a particular focus on assessing T-wave alternans, heart rate variability, and acceleration and deceleration capacity. The analysis was facilitated by the CardioScan Holter Analysis Software, specifically CardioScan 12, developed by DM Software Inc., headquartered in Beijing, China.

It is noteworthy that this analysis rigorously excluded ectopic beats or those originating from sources outside the sinus rhythm. Furthermore, individuals characterized by a high incidence of atrial and ventricular ectopic beats, specifically exceeding a frequency of more than 10 beats per hour, were deliberately excluded from the study’s participant pool. In alignment with our commitment to scientific rigor, periods marked by noise, artifacts, premature beats, and post-extrasystolic pauses underwent meticulous screening and were subsequently excluded from further investigation. In the pursuit of a comprehensive examination, we harnessed the capabilities of the SAECG device, DM System Company Ltd., Beijing, China, making use of its 3-channel (orthogonal lead) setup paired with a 7-wire recorder. The exploration into late potentials strictly adhered to a standardized protocol [41]. Similarly, the assessment of ventricular late potentials in the time domain consistently employed the CardioScan 12 software.

### 2.2. Definitions

In adherence to the guidelines outlined by the ESC, the diagnosis of HF required the manifestation of clinical symptoms such as dyspnea, fatigue, and ankle edema. This diagnosis was further substantiated by observable signs of heart failure and the objective confirmation of cardiac dysfunction through the measurement of NT-pro BNP levels equal to or exceeding 125 pg/mL, supported by echocardiographic assessment [1]. For a comprehensive assessment, a 24 h HOLTER ECG and evaluation of LVP were subjected to manual interpretation using the Cardioscan 12 software.

HRV analysis was omitted in cases where recordings exhibited more than 10% artifacts. Both time and frequency domain analyses of HRV, along with the assessment of deceleration and acceleration capacity, were automatically computed and documented [42]. The time-domain indices included parameters such as the standard deviation of RR intervals for the entire duration (SDNN; normal values below 50 ms), the standard deviation of the averages of NN intervals in each 5 min segment across the entire recording (SDANN; normal values below 40 ms), the mean of the 5 min normal-to-normal intervals throughout the complete recording (SDANN index; normal values below 30 ms), the square root of the mean of the squares of the successive differences between adjacent NN intervals (RMSSD; normal values below 15), the ratio of NN50 to the total count of NN intervals (PNN50; normal values below 0.75%), and the total count of all NN intervals divided by the height of the histogram of all NN intervals, measured on a discrete scale with bins of 7.8125 ms (triangular index) [43]. The frequency-domain indices included specific frequency bands: high frequency (hF), spanning the range of 0.15 to 0.4 Hz; low frequency (lF), covering the range from 0.04 to 0.15 Hz; and very low frequency (vlF), extending from 0.0033 to 0.04 Hz [44].

The determination of deceleration capacity (DC) and acceleration capacity (AC) was based on an innovative phase-rectified signal averaging methodology designed for the examination of quasi-periodic oscillations within noisy and non-stationary signal data. The relative counts of deceleration and acceleration sequences, each comprising 1 to 10 RR intervals, were categorized into three risk-stratified groups: low-risk (values falling between 4.5 ms and 10 ms), intermediate-risk (ranging from 2.5 ms to 4.49 ms), and high-risk (ranging from 0 to 2.49 ms), as previously described in the literature [42].

Time-domain analysis was applied to evaluate TWA across all available channels. TWA was identified as the maximum observed value within any channel, with a T-wave alternans value equal to or exceeding 60 μV considered as indicative of a positive finding.

**Figure 1:**
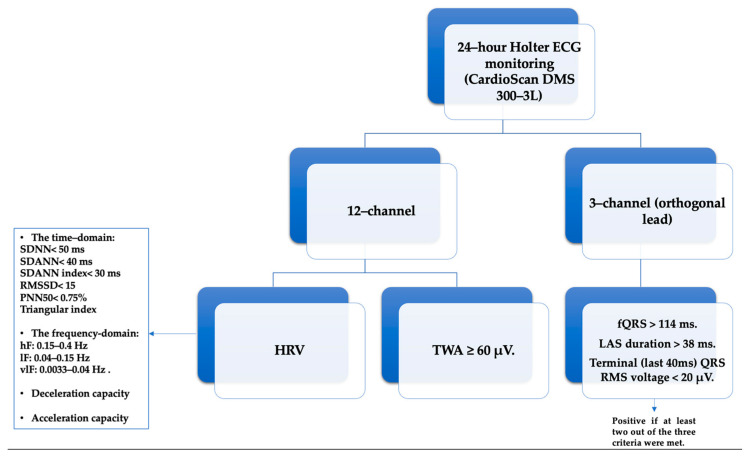
The Holter ECG/24 h parameters analyzed: HRV—Heart Rate Variability, SDNN—standard deviation of RR intervals for the entire duration, SDANN—standard deviation of the averages of NN intervals in each 5 min segment across the entire recording, SDNN index—mean of the 5 min normal-to-normal intervals throughout the complete recording, RMSSD—square root of the mean of the squares of the successive differences between adjacent NN intervals, PNN50—ratio of NN50 to the total count of NN intervals, vlF—very low frequency, lF—low frequency, hF—high frequency, TWA—T-wave alternans, (fQRS)—filtered QRS complex, LAS—low-amplitude signals, RMS—root mean square.

For the acquisition of SAECG, each study participant underwent a 3-lead resting electrocardiogram using the Holter ECG DMS 300-4L device. A total of 500 cardiac cycles were recorded, typically within a time frame of 12 to 15 min. Following this, the late potential assessment was repeated using the software integrated into the same device. The QRS waveforms underwent bi-directional filtering within the frequency range of 40–250 Hz. Ventricular late potentials were identified by analyzing the filtered QRS complex, considering criteria such as a filtered QRS complex duration (fQRS) exceeding 114 milliseconds, the presence of low-amplitude signals (LAS) lasting more than 38 milliseconds within the terminal portion of the QRS complex, and a root mean square (RMS) voltage within the terminal 40 milliseconds falling below 20 microvolts (μV). A diagnosis of ventricular late potentials was confirmed if at least two out of the three criteria were met [45,46] (Figure 1).

In the examination, DM was defined as a condition characterized by a morning-fasting glucose level equal to or exceeding 126 mg/dL, a glycated hemoglobin (HbA1c) level of 6.5% or higher, or the use of antidiabetic medications. For statistical analysis, the parameter utilized was the HbA1c concentration. Hyperuricemia was defined as an elevated serum urate concentration exceeding 6 mg/dL in females and 7 mg/dL in males. CKD was established following the Kidney Disease Outcomes Quality Initiative guidelines, indicating impairment in renal function with a glomerular filtration rate (GFR) of less than 60 mL/min per 1.73 m^2^, the presence of kidney damage markers, or both, persisting for at least 3 months, irrespective of the underlying etiology [47]. Body Mass Index (BMI) was calculated by dividing an individual’s weight in kilograms by the square of their height in meters [43].

### 2.3. Statistical Analysis

Utilizing version 4.3.2 of the R programming language, the statistical analysis commenced with an assessment of data distribution via the Shapiro–Wilk test. For normally distributed continuous variables, the presentation involved mean ± standard deviation (SD). Conversely, non-normally distributed continuous variables were summarized descriptively using the median and interquartile range (IQR: 25–75%). Categorical variables were depicted through frequencies and percentages. Group differences were evaluated employing the independent samples *t*-test for normally distributed variables and the Mann–Whitney U test for variables deviating from normal distribution. To explore the correlation between parameters, Pearson’s correlation coefficient (Pearson’s r) was employed, while instances not meeting parametric assumptions utilized Spearman’s rank correlation coefficient (Spearman’s ρ) to elucidate the relationship between variables. Categorical group comparisons were conducted through the chi-squared test. The threshold for statistical significance was set at a *p*-value of less than 0.05 (*p* < 0.05).

## 3. Results

### 3.1. Baseline Characteristics

This research involved a cohort of 60 patients, with 25% being female, all diagnosed with Heart Failure, with an LVEF < 50% of ischemic etiology. The mean age of the study population was 66 ± 11 years, and they presented with various concomitant medical conditions, which will be detailed in this investigation. Specifically, 32% of the patients were identified as having diabetes (*n* = 19), 25% were diagnosed with chronic kidney disease (*n* = 15), 37% exhibited hyperuricemia (*n* = 22), and a significant 77% (*n* = 46) were classified as overweight or obese. It is important to note that, as previously explained, comprehensive blood test analyses indicated the absence of anemia, infection, electrolyte imbalances, and showed normal liver and thyroid functions among the patients. Additionally, none of the patients were using medications known to induce arrhythmogenic effects. Subsequently, we present the comparative analysis of selected echocardiographic parameters between patients with and without diabetes (Table 1), obesity, CKD, and hyperuricemia.

Within the realm of our academic inquiry, it is pertinent to bring attention to the findings derived from our analysis employing PW tissue Doppler imaging. Notably, a substantial reduction in systolic myocardial velocity (S′) values (*p* = 0.005) was unveiled in the cohort of patients diagnosed with diabetes (Table 1). Additionally, it is crucial to underscore the conspicuous abbreviation of the MV deceleration time in the same group (*p* = 0.006).

In the context of other conventional echocardiographic parameters, no statistically significant disparities were detected between the two study groups regarding the left ventricular diastolic dimension, left ventricular systolic and diastolic volumes, the ejection fraction, E/A ratio, E/e′, mitral annular plane systolic excursion (MAPSE), cardiac output, or left atrium area. In our investigation of echocardiographic parameters among individuals with and without obesity, notable distinctions were observed in the left ventricular diastolic diameters (58.0 (53.3–63.8) mm vs. 51.0 (48.3–52.0) mm; *p* = 0.002) and there was a marginal difference in the left ventricular diastolic volumes (211.0 (175.0–263.0) mL vs. 160.0 (137.0–222.0) mL; *p* = 0.058). Conversely, in the context of additional conventional echocardiographic parameters, no statistically significant variances were discerned between the cohorts of individuals with and without obesity regarding the left ventricular systolic volume (136.0 (121.0–179.0) mL vs. 101.0 (89.0–150.0) mL; *p* = 0.062), ejection fraction (31.5 ± 9.4% vs. 31.9 ± 10.4%; *p* = 0.908), E/A ratio (1.0 (0.7–1.6) vs. 0.8 (0.6–1.7); *p* = 0.529), E/e′ (11.1 (8.0–14.7) vs. 12.1 (10.8–18.4); *p* = 0.368), MAPSE (12.2 ± 2.4 mm vs. 12.9 ± 3.8 mm; *p* = 0.405), cardiac output (5.09 ± 1.31 L/min vs. 4.50 ± 1.49 L/min; *p* = 0.162), or left atrium area (22.7 (18.3–27.9) cm^2^ vs. 20.5 (16.0–24.8) cm^2^; *p* = 0.165).

Unlike the first two comorbidities under investigation, CKD and hyperuricemia did not exhibit statistically significant changes in echocardiographic parameters. Henceforth, there were no discernable statistically significant discrepancies identified between the cohorts of subjects afflicted with and without CKD concerning the left ventricular diastolic dimension (57.0 (51.5–59.0) mm vs. 56.0 (51.0–64.0) mm; *p* = 0.388), left ventricular systolic volume (129.0 (117.0–152.0) mL vs. 136.0 (96.0–212.0) mL; *p* = 0.326) and diastolic volume (181.0 (172.0–205.0) mL vs. 215.0 (165.0–278.0) mL; *p* = 0.109), ejection fraction (29.8 ± 10.1% vs. 32.3 ± 9.5%; *p* = 0.389), E/A ratio (1.0 (0.5–1.7) vs. 0.9 (0.7–1.7); *p* = 0.663), E/e′ (11.3 (8.9–19.9) vs. 11.7 (8.0–14.5); *p* = 0.322), MAPSE (11.6 ± 2.6 mm vs. 12.6 ± 2.8 mm; *p* = 0.247), cardiac output (4.5 ± 1.4 L/min vs. 5.0 ± 1.3 L/min; *p* = 0.232), or left atrium area (23.1 (17.8–25.7) cm^2^ vs. 20.7 (18.3–26.5) cm^2^; *p* = 0.778).

Moreover, within the cohorts of subjects with and without hyperuricemia, no statistically significant differences were noted in relation to the aforementioned parameters: the left ventricular diastolic dimension (56.5 (49.5–60.0) mm vs. 55.5 (51.3–63.0) mm; *p* = 0.612), left ventricular systolic volume (153.0 (122.0–178.0) mL vs. 131.0 (95.3–173.0) mL; *p* = 0.290) and diastolic volume (213.0 (182.0–254.0) mL vs. 195.0 (164.0–263.0) mL; *p* = 0.514), ejection fraction (29.4 ± 8.7% vs. 33.0 ± 10.0%; *p* = 0.168, E/A ratio (1.2 (0.6–2.3) vs. 0.9 (0.7–1.5); *p* = 0.395), E/e′ (11.9 (9.6–15.8) vs. 11.1 (8.0–16.4); *p* = 0.365), MAPSE (12.4 ± 2.3 mm vs. 12.3 ± 3.0 mm; *p* = 0.845), cardiac output (5.3 ± 1.4 L/min vs. 4.7 ± 1.2 L/min; *p* = 0.137), or left atrium area (25.2 (18.5–28.0) cm^2^ vs. 20.4 (17.5–25.5) cm^2^; *p* = 0.147).

### 3.2. Holter ECG Parameters

Our study subsequently advanced to perform comparative assessments between the mentioned patient characteristics and the parameters obtained from a 24 h Holter ECG monitoring phase. This segment of our inquiry involved a thorough examination of Holter ECG parameters in association with various comorbidities. Specifically, we investigated measures of heart rate variability, including the SDNN, SDANN, SDNN index, RMSSD, PNN50, Triangular index, VLF, LF, and HF, along with the acceleration and deceleration capacity. Additionally, parameters related to TWA and LVP were scrutinized. Table 2 presents average NT-proBNP values and the NYHA class distribution for our patient cohort. In Table 2, data are provided for the entire study cohort and subgroups of patients, categorized into those with and without diabetes. Normally distributed data were expressed as mean values with corresponding standard deviations and subjected to statistical comparison using the independent samples *t*-test. Non-normally distributed data were represented as median values along with the interquartile range, and comparisons were conducted utilizing the Mann–Whitney U test. Differences in categorical variables were assessed using the chi-squared test.

In our study cohort, 55% of patients, specifically 33 individuals, experienced dyspnea categorized under NYHA class II, while 35% (21 patients) were classified under NYHA class III. A minor percentage of 7% (four patients) had NYHA class I, and only 3% (two patients) were identified under NYHA class IV. The mean NT-proBNP level in our study cohort was quantified at 2568 pg/mL, with values spanning a range from 634 to 6193 pg/mL. Both TWA and LVP exhibited no statistically significant differences between patients with diabetes and those without diabetes, as indicated by *p*-values of 0.405 and 0.221, respectively.

Unexpectedly, there was a notable resemblance in heart rate variability parameters between the two groups, despite the well-documented impact of diabetes on the autonomic nervous system. However, we did identify a statistically significant correlation between diabetes and the Triangular index (*p* = 0.035). Hence, the inquiry arises as to whether a diminished Triangular Index, in contrast to other established HRV parameters, exhibits an association with an elevated risk of cardiovascular mortality and might represent the foremost modifiable parameter for HRV. This consideration is pertinent given that the Triangular Index is comparatively less scrutinized in comparison to conventional HRV parameters. In the context of the acceleration and deceleration capacity within our study cohort, we did not observe any statistically significant differences in the acceleration capacity. However, a robust and statistically significant association was established between diabetes and the deceleration capacity (*p* = 0.002), an important aspect that will be discussed later.

In Table 3, we have outlined the existence of additional comorbidities, namely obesity, CKD, and hyperuricemia. To explore the associations and correlations between these comorbidities and pertinent variables, including creatinine clearance, BMI, and uric acid levels, a correlation analysis was conducted.

Within the realm of creatinine clearance, the most prominent modifications in HRV parameters were observed, showcasing a statistically significant positive correlation with HRV measures. This correlation was evident with RMSSD (*p* = 0.026, Spearman’s ρ = −0.287), PNN50 (*p* = 0.013, Spearman’s ρ = −0.318) and, in relation to the frequency domain parameters, a correlation was identified with high-frequency (hF) power (*p* = 0.026, Spearman’s ρ = −0.287).

Significantly, our investigation did not reveal substantial alterations in HRV parameters when comparing patients with obesity to those without obesity.

Regarding the association between uric acid levels and distinct Holter ECG patterns, our analysis brought to light statistical significance concerning the deceleration capacity (*p* = 0.045, Pearson’s r = −0.260). It is noteworthy that, although not reaching the same level of statistical significance, a notable correlation was observed with the acceleration capacity (*p* = 0.078, Spearman’s ρ = 0.229), a finding we consider relevant to report due to its potential clinical significance.

In the case of LVP and TWA, the data exhibited a non-normal distribution and, consequently, were presented as the median (interquartile range). Group comparisons were conducted using the Mann–Whitney U test. The analysis, depicted in Table 4 and Table 5, demonstrated a lack of statistically significant associations between the examined parameters and the comorbidities under investigation.

### 3.3. Examination of Statistically Significant Parameters

**Figure 2:**
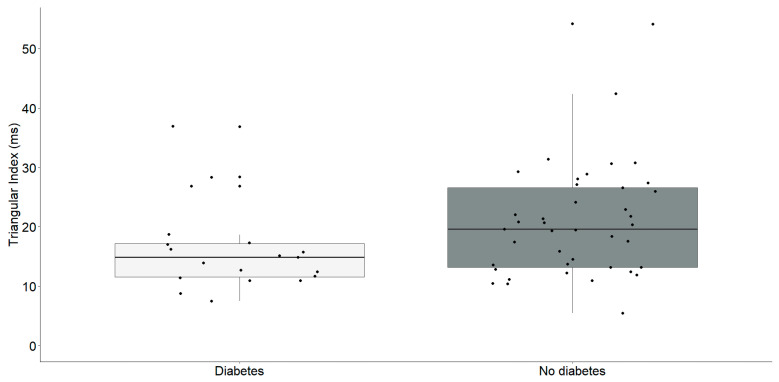
Triangular index (ms) differences between chronic heart failure patients with (*n* = 19) and without diabetes (*n* = 41). The interquartile range is depicted by the rectangular box, with the first quartile (Q1, 25%) and the third quartile (Q3, 75%) represented as the bottom and top boundaries, respectively. The median value of the dataset is indicated by the horizontal line inside the box, and each dot corresponds to an individual datapoint.

**Figure 3:**
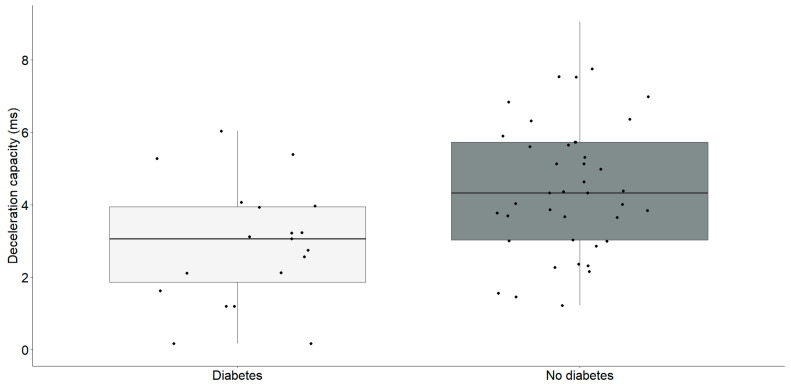
Deceleration capacity (ms) differences between chronic heart failure patients with (*n* = 19) and without diabetes (*n* = 41). The interquartile range is depicted by the rectangular box, with the first quartile (Q1, 25%) and the third quartile (Q3, 75%) represented as the bottom and top boundaries, respectively. The median value of the dataset is indicated by the horizontal line inside the box, and each dot corresponds to an individual datapoint.

Our findings revealed a significant reduction in the Triangular Index and deceleration capacity in individuals with diabetes, as illustrated in the Table 2. Subsequently, our investigation aimed to perform a focused analysis to clarify the specific correlations between these parameters, as outlined in Figure 2 and Figure 3.

Individuals identified with diabetes (Median: 14.9, IQR: 11.6–17.2) exhibited a notably reduced triangular index in comparison to those without diabetes (Median: 19.6, IQR: 13.2–26.6) with statistical significance (U = 256, *p* = 0.035).

Participants diagnosed with diabetes (2.9 ± 1.6) exhibited a significantly lower deceleration capacity compared to individuals without diabetes (4.5 ± 1.8) (t = −3.23, df = 58, *p* = 0.002).

**Figure 4:**
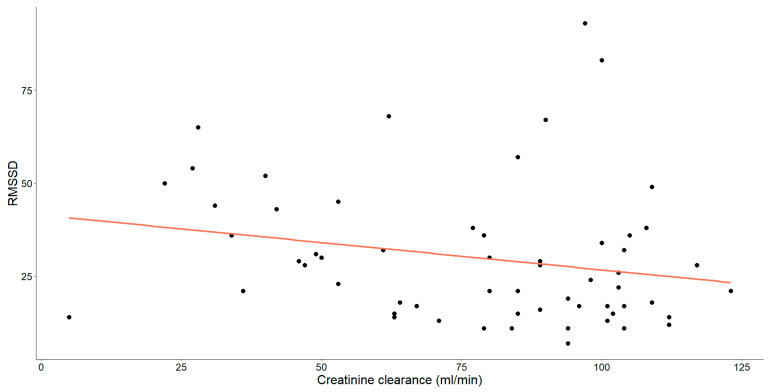
The correlation between creatinine clearance level (mL/min) and RMSSD in patients with chronic heart failure is illustrated. Each data point represents an individual and the orange line depicts the best-fit line or regression line.

**Figure 5:**
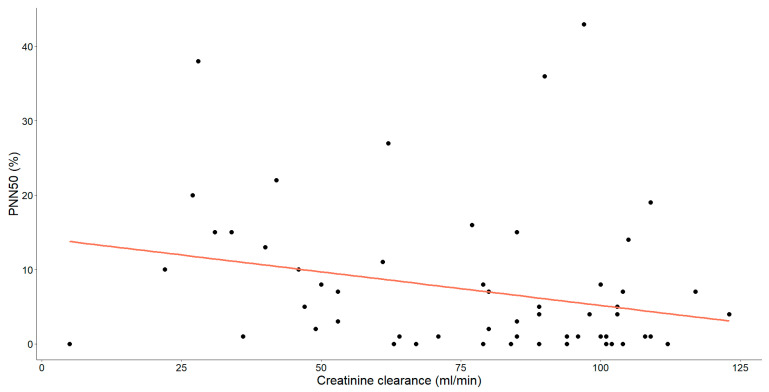
The association between creatinine clearance levels (mL/min) and PNN50 (%) in patients with chronic heart failure is illustrated. Each data point corresponds to an individual and the orange line signifies the regression line or line of best-fit.

**Figure 6:**
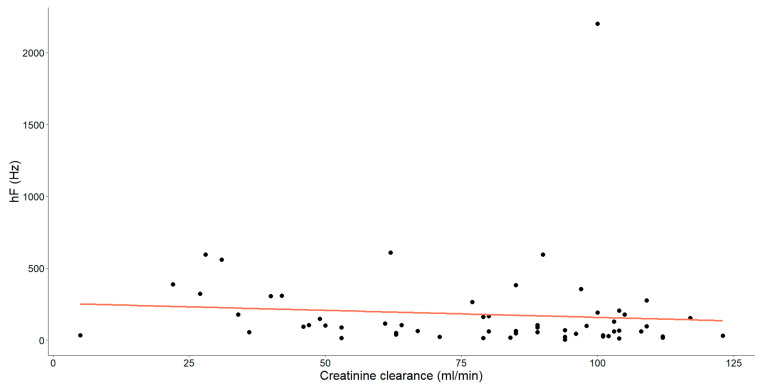
The association between creatinine clearance level (mL/min) and high frequency (hF, Hz) in chronic heart failure patients is illustrated. Each dot signifies an individual data point and the orange line depicts the best-fit line (regression line).

As mentioned earlier, our study unveiled substantial correlations between creatinine clearance and various HRV parameters. Specifically, those showing statistical significance have been thoroughly examined and are detailed in Figure 4, Figure 5 and Figure 6.

A statistically significant, weak-to-moderate negative correlation was noted between RMSSD and creatinine clearance levels (Spearman’s ρ = −0.287, *p* = 0.026).

Furthermore, a statistically significant, moderate negative relationship was observed between PNN50 and creatinine clearance (Spearman’s ρ = −0.318, *p* = 0.013).

Finally, a statistically significant negative association, characterized as weak-to-moderate (Spearman’s ρ = −0.287, *p* = 0.026), was identified between these variables.

**Figure 7:**
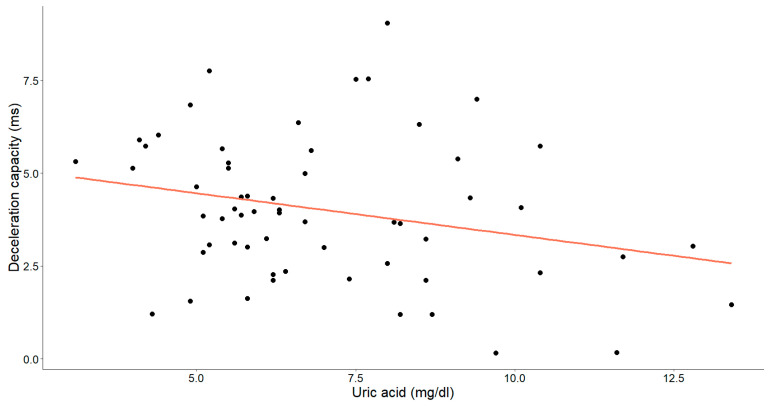
The association between uric acid concentrations (mg/dL) and deceleration capacity (ms) in patients with chronic heart failure is depicted in the graph. Each data point corresponds to an individual and the orange line signifies the best-fit regression line.

**Figure 8:**
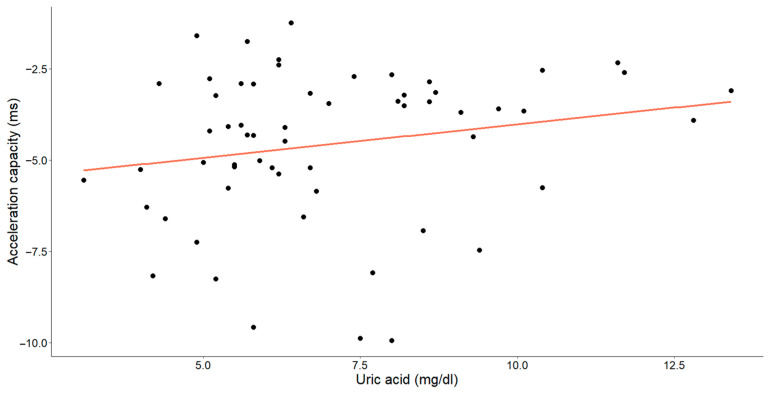
The correlation between uric acid concentrations (mg/dL) and acceleration capacity (ms) in patients with chronic heart failure is illustrated in the graph. Each data point corresponds to an individual and the orange line denotes the best-fit regression line.

In assessing the efficacy of uric acid levels, our objective was to emphasize the supplementary value of the acceleration and deceleration capacity (Figure 7 and Figure 8).

A statistically significant, weak-to-moderate inverse relationship was identified between the uric acid levels and deceleration capacity (Pearson’s r = −0.260, *p* = 0.045). Additionally, a weak-to-moderate positive relationship between uric acid and the acceleration capacity was observed (Spearman’s ρ = 0.229, *p* = 0.078), with the *p*-value approaching, but slightly exceeding, the threshold for statistical significance.

## 4. Discussion

Significant contributors to both mortality and morbidity, ischemic cardiomyopathy and HF, along with comorbidities, impose substantial healthcare expenditures. In developed nations, the age-adjusted incidence of heart failure may exhibit a declining trend, potentially attributed to the enhanced management of cardiovascular diseases. However, the overall incidence is on the rise, primarily due to the aging demographic [48,49].

Despite the imperative role played by imaging techniques in enhancing structural characterization, the diagnosis of HF requires the confluence of clinical symptoms and/or signs indicative of HF alongside objective evidence of cardiac dysfunction [1]. Modern Holter monitoring, often considered secondary in the diagnosis of HF or ischemic cardiomypathy, reveals its potential as an invaluable instrument for investigating the intricate factors contributing to the mechanisms of sudden death [13]. Therefore, while continuous ambulatory Holter ECG monitoring has been traditionally relegated in conventional wisdom, it also plays a crucial role in risk stratification [50,51].

As the global populace ages, there is a noticeable surge in the prevalence of non-cardiovascular comorbidities impacting individuals with HF, including those contending with ischemic heart failure [52,53]. The explicit consideration of ischemic heart failure in this context introduces an additional layer of complexity, given its unique etiological factors and potential interplay with non-cardiac comorbidities. Ischemic heart failure, frequently arising from coronary artery disease, presents a distinct set of challenges in both research and clinical practice [38,54]. It is imperative to underscore that a common thread weaving through a majority of non-cardiac comorbidities linked to HF lies in their inherent capacity to potentially induce left ventricular dysfunction and precipitate HF [40,55,56]. A comprehensive and all-encompassing approach, reflective of the intricacies observed in clinical practice, is indispensable for advancing our understanding of HF pathophysiology and optimizing patient care in the realm of ischemic heart failure and its concurrent non-cardiac comorbidities [37,56,57].

The results obtained from our investigation indicated that specific comorbidities lead to an augmentation of certain less conventional Holter ECG parameters related to HRV, even though other parameters maintain normal values. In the context of our study, the triangular index and the acceleration and deceleration capacity exhibited statistically significant deviations from normal values in specific comorbid conditions under scrutiny (e.g., diabetes and hyperuricemia).

Our objective was to elucidate the initial impact on the deceleration capacity and explore its potential prognostic significance. The autonomic nervous system consists of the sympathetic and parasympathetic branches. Dysregulation in the balance of these branches can lead to a decrease in the deceleration capacity. Specifically, a reduction in the parasympathetic (vagal) tone may hinder the heart’s ability to decelerate efficiently after exposure to a stressor or stimulus, resulting in a diminished deceleration capacity [58,59]. Simultaneously, the activity of the sympathetic system may remain within normal parameters, preserving the acceleration capacity [58,60].

Regarding the first comorbidity that we studied, specifically diabetes, the disruption of the autonomic nervous system influences heart electrical stability, potentially causing changes in TWA, VLPs, and HRV [57,58,60]. Fluctuations in blood glucose levels, chronic inflammation, oxidative stress, insulin resistance, and metabolic syndrome further contribute to disturbances in heart rhythm [56,58]. It is crucial to note that diabetes can also induce structural and functional alterations in the heart, referred to as diabetic cardiomyopathy. These alterations create an environment within the heart that promotes arrhythmogenic potential [37,57,59]. In our study, individuals diagnosed with diabetes (2.9 ± 1.6) exhibited a notably diminished deceleration capacity compared to those without diabetes (4.5 ± 1.8), and this difference was found to be statistically significant (t = −3.23, df = 58, *p* = 0.002).

Similar to our study, Wang et al. found that the deceleration capacity is significantly reduced in individuals with diabetes, serving as a distinct indicator for identifying those with pronounced autonomic nervous system impairment. Identifying individuals at an elevated risk of sudden death is crucial for timely clinical intervention and treatment, thereby mitigating or circumventing the deleterious consequences associated with autonomic neuropathy. This marker, reflecting a compromised quality of life in individuals with congestive heart failure and diabetes, underscores the importance of early preventative measures and therapeutic interventions [61]. An additional HRV parameter found to be altered in patients with diabetes is the triangular index. According to Hammerle et al., their study suggests that the triangular index may serve as a predictive indicator for cardiovascular mortality [62]. The modification in the triangular index indicates a change in the geometric configuration of the Poincaré plot, a widely used graphical representation in HRV analysis. Specifically linked to the dispersion and distribution of successive interbeat intervals, the triangular index provides insights into the modulation of heart rate dynamics by the autonomic nervous system [62,63,64].

In reference to the next comorbidity, hyperuricemia, elevated uric acid levels are frequently associated with systemic inflammation and oxidative stress, both of which carry implications for the cardiac electrophysiological profile [65,66,67,68,69,70,71,72,73,74,75,76,77,78]. Individuals afflicted by metabolic syndrome, a cluster of risk factors for cardiovascular disease, often exhibit heightened uric acid levels, concurrently linked to occurrences of LVP, TWA, and reduced HRV [37,55,56,57]. In certain cases, uric acid crystals may accumulate in diverse tissues, including the heart, inducing inflammation and tissue injury. Though less common, these deposits have the potential to influence the heart’s electrical properties [68,71,72]. As mentioned earlier, it is noteworthy that both the acceleration and deceleration capacity showed aberrations in individuals with elevated uric acid levels. Particularly, there is a robust statistical significance observed in the deceleration capacity, while the acceleration capacity demonstrated a marginally significant association.

In our investigation, noticeable modifications were identified in either the triangular index or deceleration capacity among patients exhibiting diabetes or hyperuricemia. Importantly, these alterations were not apparent in other frequently employed parameters delineating Heart Rate Variability (HRV). Given the statistically significant nature of these identified parameters, which have not been comprehensively explored, we advocate for forthcoming investigations to systematically scrutinize these parameters. This recommendation is based on the potential of these parameters to serve as early indicators of autonomic nervous system dysregulation, thereby facilitating timely identification and intervention in affected individuals.

Concerning the third comorbidity, respectively, CKD, it has the potential to induce fluid retention and disturbances in the electrolyte equilibrium, particularly affecting potassium levels. Variations in potassium levels may disrupt cardiac electrophysiology, leading to the onset of arrhythmias. CKD is marked by the accumulation of uremic toxins within the body, resulting in multifaceted consequences for the cardiovascular system [72,73,74]. These toxins can stimulate myocardial fibrosis, trigger inflammation, and induce oxidative stress, collectively impacting the heart’s electrical properties [39,74,75].

Our findings revealed a statistically significant association between CKD and the parameters of RMSSD, pNN50, and HF in HRV analysis. The elevated values of these parameters suggest increased parasympathetic activity, indicating a potential prevalence of vagal (parasympathetic) tone [20,21]. Specifically, RMMSD and pNN50 signify distinct modifications in the short-term variability of heart rate under the influence of the parasympathetic nervous system. In contrast, high-frequency power is typically associated with respiratory sinus arrhythmia, reflecting the influence of the vagus nerve on heart rate dynamics [19,24]. In their investigation, Avula et al. concluded that CKD has the potential to impact HRV parameters, with statistical significance identified specifically in the case of SDNN and SDANN [74]. The modification of SDNN and SDANN in their investigation could be attributed to the elevated representation of individuals in CKD stages IV and V, characterized by a heightened prevalence of anemia. Such anemia may selectively impact the overall measure of HRV without exerting a discernible influence on the respiratory modulation of heart rate. These parameters exhibited statistical significance in the study conducted by Kida et al. Furthermore, they concluded that altered HRV in patients with CKD may predict major adverse cardiovascular events [75,76].

As for the last comorbidity, namely obesity, it is intricately linked to chronic low-grade inflammation, potentially inducing an imbalance in the autonomic nervous system and correlating with insulin resistance and metabolic syndrome [75,76,77]. Additionally, obesity demonstrates a heightened prevalence of obstructive sleep apnea, a condition acknowledged for its detrimental impact on HRV, LVPs, or TWA [43,44]. The increased deposition of adipose tissue around the heart can disrupt the conduction system, fostering an environment conducive to arrhythmias [44]. Moreover, the physical presence of excess body fat may exert compressive effects on the chest, hindering the heart’s optimal expansion and contraction, thereby potentially influencing HRV [44,79].

In contrast to previous investigations establishing an association between sympathovagal imbalances and BMI in HF patients, our study revealed a lack of correlation between BMI and HRV indices [80,81]. Certain limitations inherent to our study design may contribute to this circumstance, including the limited representation of lean individuals in our cohort. Furthermore, the study relied on BMI as an indicator of obesity without assessing key factors such as body composition (body fat, muscle, and water composition) or body-fat distribution. The distribution of adipose tissue, especially regarding visceral adiposity, is recognized for its potential to yield distinct effects on autonomic function. Notably, central obesity has been linked to modifications in HRV [44]. Additionally, variations in individual responses to obesity and its metabolic repercussions are conceivable. The manifestation of changes in HRV may depend on factors such as genetic predisposition, lifestyle choices, and overall health status [43,44,76]. However, our investigation aligns with others in observing a lack of statistical disparity between BMI and HRV, as evidenced in the results by Tacoy et al. [43]. Yadav et al. further demonstrated that BMI exhibits a tenuous correlation with cardiac autonomic markers of HRV. However, an elevated waist–hip ratio exhibited a robust association with diminished cardiac parasympathetic activity and heightened sympathetic activity in individuals characterized as obese [44]. Therefore, future investigations into autonomic function should not singularly focus on BMI, but also consider the Waist–Hip Ratio for a comprehensive assessment.

During our investigation, no statistically significant associations were observed between TWA or LVP and any comorbidities. This phenomenon may be attributed to diverse factors. Variations in study outcomes may arise from dissimilarities in patient cohorts, including discrepancies in the duration and severity of underlying comorbidities. Conditions such as diabetes and CKD are acknowledged for their impact on the autonomic nervous system, potentially affecting the absence of TWA or LVP [58,73,74]. Additionally, the utilization of medications for diverse conditions associated with diabetes, such as beta-blockers, particularly in HF cases within our cohort, introduces an additional dimension due to their antiarrhythmic properties, potentially influencing the expression of TWA and LVP [58,79].

In the context of TWA and LVP, the research on the influence of beta blockers is limited, contrasting with their well-established role in HRV. Beta blockers play a pivotal role in modulating HRV by antagonizing beta-adrenergic receptors, thereby attenuating the sympathetic nervous system’s impact on cardiac dynamics. This mechanistic intervention induces a more uniform and diminished heart rate, resulting in a noticeable reduction in the amplitude of HRV. The observed phenomenon is explained by the heightened predominance of the parasympathetic nervous system following beta blocker administration, tempering the physiological oscillations intrinsic to normal heart rate fluctuations. In our investigation, the entire patient cohort received beta blocker therapy as a fundamental component of their therapeutic regimen mandated by the prevailing pathologies characterized by HF [82,83]. Future research inquiries should systematically explore potential correlations between heart rate variability and nuanced parameters such as beta blocker dosage and specific beta blocker classifications. This analytical trajectory promises to provide a comprehensive understanding of the intricate interplay between beta blocker pharmacotherapy and cardiac autonomic regulation [82].

However, a worse outcome is not solely predicted by diminished heart rate; augmenting heart rate variability is also associated with an unfavorable prognosis. While augmented HRV is commonly regarded as an indicator of autonomic nervous system adaptability and cardiovascular well-being, its interpretation is nuanced as there are circumstances wherein increased HRV may not be advantageous and could signify underlying health issues. This variability in HRV responses can be attributed to various factors, including individual variations in normal HRV values influenced by age, fitness levels, and overall health [84]. For example, instances of heightened sympathetic nervous system activity or acute stressors may lead to increased HRV, which, in the context of chronic stress or sympathetic overactivity, may indicate autonomic dysregulation, portraying a compensatory response suggestive of persistent stress rather than an adaptive state [85]. Moreover, elevated HRV in the presence of specific cardiac arrhythmias or conduction disorders may reflect increased rhythm variability without necessarily indicating improved cardiac function. Medical conditions such as cardiac dysautonomia, prevalent in certain neuropathic conditions or autonomic dysfunction disorders, may manifest as increased HRV. In metabolic conditions like diabetes or metabolic syndrome, augmented HRV might be associated with autonomic neuropathy resulting from nerve damage affecting heart rate regulation. Hyperthyroidism, characterized by excessive thyroid hormone levels, can contribute to increased HRV, emphasizing the delicate balance between thyroid function and cardiovascular health. Notably, in athletes undergoing excessive endurance training or overtraining, heightened HRV may signify physiological stress on the body, potentially associated with adverse cardiovascular outcomes. Lastly, the age-related decline in HRV is noteworthy, particularly in older individuals, where increased HRV may not necessarily align with expectations of cardiovascular advantages [23,84,85].

Despite our obtained results, it is crucial to recognize that several investigations propose an inverse relationship between the severity of coronary artery disease and HRV. This suggests that the degree of coronary ischemia may exert a discernible influence on HRV. Compromised perfusion to the myocardium triggers sympathetic nervous system activation, resulting in an augmented release of stress mediators, notably, adrenaline. This sympathoexcitation leads to an elevation in the heart rate and concurrently may contribute to the attenuation of HRV. Additionally, it is noteworthy that the anatomical localization of ischemic events within the cardiac milieu may introduce nuanced modulations to autonomic function, imparting heterogeneity in the relationship between ischemia and HRV across distinct cardiac regions [82,83,86]. Prospective investigations involving larger cohorts should systematically incorporate an evaluation of both the magnitude of ischemic involvement and the specific anatomical localization of coronary artery disease.

Although our study did not evaluate long-term adverse cardiovascular outcomes, numerous investigations have established their predictive capacity for significant adverse cardiovascular events [58,71,77,78,79]. To address this limitation, we intend to incorporate data on long-term cardiovascular events and extended follow-up in future investigations.

In considering future prospects, the utilization of Holter ECG monitoring for individuals with ischemic cardiomyopathy and HF, coupled with emerging comorbidities, has the potential not only to refine risk stratification, but also to guide early therapeutic interventions, contributing to an improvement in the overall quality of life. Presently, there exists no universally accepted risk scoring system specifically tailored for the determination of defibrillator implantation suitability in heart failure patients, and none of the existing scoring systems incorporate HRV as a primary parameter [87,88]. The LVEF assumes paramount significance in the decision-making process regarding eligibility for defibrillator implantation, with LVEF values at or below a commonly accepted threshold of 35%, signifying an indication for primary prevention. However, the risk assessment in this context is often characterized by its multifactorial nature. Guidelines extend their considerations beyond LVEF, incorporating additional variables such as the NYHA functional class, NT-proBNP levels, age, comorbidities, and medication usage. Furthermore, the underlying etiology of heart failure, whether ischemic or non-ischemic, plays a pivotal role in prognosis, with ischemic heart disease being potentially associated with a heightened risk of adverse events [1].

The pre-eminent long-term intervention for averting sudden cardiac death (SCD) in individuals at high risk is the utilization of an implantable cardioverter defibrillator (ICD). The efficacy of ICD implementation is contingent upon a thorough risk assessment [1]. Consequently, the decision to implant an ICD should be reserved for individuals demonstrating a sustained and enduring high risk of SCD, as opposed to those presenting with a potentially reversible SCD risk. In instances where there is a transient risk for SCD, individuals may be considered suitable candidates for a wearable cardioverter defibrillator (WCD). The WCD also serves as a viable alternative for individuals awaiting ICD implantation or those following ICD explanation, such as cases related to infections or endocarditis [89].

A pivotal instrument in risk stratification is the Seattle Heart Failure Model, an extensively applied prognostic model that assimilates a spectrum of clinical, laboratory, and medication-related variables. This model elucidates estimates for both one-year and five-year mortality risks among individuals afflicted by heart failure. Its constituent elements encompass demographic attributes (age, sex), physiological parameters (blood pressure, heart rate), functional categorization (NYHA functional class), pharmaceutical interventions, and salient laboratory metrics [90].

Certain contemporary ICDs feature automated algorithms designed to furnish comprehensive daily insights into HF conditions. These algorithmic tools contribute to elucidating the bidirectional causative mechanisms between HF and ventricular arrhythmias, offering potential in discerning predisposing factors. An illustrative case is found in the Multisensor Chronic Evaluation in Ambulatory Heart Failure Patients (MultiSENSE) study, where a novel HF monitoring algorithm, namely the HeartLogic index developed by Boston Scientific, was employed [87,88]. This index amalgamates physiological data derived from multiple sensors integrated into the ICD platform. The findings of the study demonstrated that the HeartLogic index serves as a sensitive and timely predictor of imminent HF decompensation. The algorithm amalgamates data from diverse sensors within the implantable device, capturing heart sounds, thoracic impedance, heart rate variability, the respiration rate, and activity levels. By perpetually monitoring these parameters, HeartLogic establishes individualized baseline values and scrutinizes deviations to identify patterns indicative of shifts in the heart failure status [88,91]. Upon detecting noteworthy deviations from baseline values, HeartLogic generates alerts, prompting healthcare providers to assess the patient’s condition and adjust treatment strategies accordingly [90,91]. For enhanced accessibility, many implantable devices featuring HeartLogic support remote monitoring. This functionality allows healthcare providers to routinely access patient data without necessitating in-person visits, enabling timely intervention when warranted. The incorporation of the time-domain analysis of HRV through remote monitoring may serve to identify patients at an elevated risk of lethal arrhythmic events and predict the occurrence of such events [91,92]. Despite the ongoing investigation and limited integration of novel Holter ECG parameters into studies, their inclusion within a comprehensive research framework undoubtedly broadens the landscape of information related to long-term prognostic assessments.

### Limitations of the Study

The primary limitation stems from the study’s single-center design and the relatively small patient enrollment. However, it is crucial to note the meticulous application of comprehensive exclusion criteria, aimed at investigating various Holter ECG parameters while minimizing the impact of concurrent comorbidities. Expanding the study to a larger patient cohort would facilitate multivariable regression analysis and the development of a multi-parameter risk stratification score. This pioneering study explores the intricate interplay between Holter ECG parameters and diverse non-cardiovascular comorbidities, paving the way for future investigations into additional comorbidities, both cardiac and non-cardiac. The Holter ECG serves as a commendable instrument for the perpetual monitoring of cardiac activity; nevertheless, its routine application encounters pragmatic constraints, especially in the analysis of specific parameters, as explicated earlier. Mitigating these limitations may necessitate advancements in technological infrastructure, the refinement of healthcare professional training, or the innovation of more user-accessible analytical tools. Notably, patients with HFpEF were not included, warranting exploration in future studies. The statistical methodology considered variables like BMI, uric acid, HbA1c, and GFR in examining correlations between comorbidities and Holter ECG parameters. Future research should employ more sophisticated statistical models, incorporating potential confounding factors like NTproBNP and LVEF. While the assessment of the ejection fraction is crucial for prospective investigations, our study did not find statistical significance in the comparison of groups based on each comorbidity, particularly regarding LVEF. An additional limitation is the omission of considerations regarding the duration of patients’ diabetes mellitus and the potential development of complications, such as diabetic cardiomyopathy. It is essential to highlight that our study cohort, primarily consisting of chronic heart failure patients, encompasses various disease stages, introducing heterogeneity that should be considered in future investigations. Lastly, the application of heart failure algorithms within defibrillators, notwithstanding the inclusion of robust parameters, has exhibited variable success in substantiating enhanced survival among patients afflicted with heart failure across diverse clinical trials. This underscores the imperativeness for additional research endeavors aimed at comprehending the intricate determinants that influence the efficacy of these algorithms, with a view to potentially refining their design or implementation strategies [87,88].

## 5. Conclusions

Our investigation aimed to extend the clinical applicability of Holter ECG beyond patients with ischemic cardiomyopathy to those with heart failure and diverse comorbidities, positioning it as a diagnostic and prospective prognostic tool. Less-utilized Holter ECG parameters, including the triangular index and the acceleration and deceleration capacity, exhibited significant diagnostic utility, particularly when conventional HRV parameters were within normal ranges.

Notably, obesity showed no discernible association with clinically modifiable ECG parameters, emphasizing the potential significance of measuring the waist–hip ratio over BMI in understanding cardiovascular implications. Furthermore, specific non-cardiac comorbidities, such as diabetes, hyperuricemia, CKD, or obesity, did not affect TWA and LVP in HF individuals. This suggests a potential influence of HF medications on these parameters, warranting further investigation.

These findings underscore the importance of non-traditional Holter ECG parameters in risk stratification for patients with various non-cardiac comorbidities, suggesting a transformative approach in incorporating these parameters as prognostic tools for diverse patient populations. However, comprehensive multicenter studies are essential to validate the long-term prognostic implications.

## Figures and Tables

**Table 1:** Comparison of echocardiographic measures in individuals with and without diabetes.

Characteristic	All Sample (*n* = 60)	Patients with Diabetes (*n* = 19)	Patients without Diabetes (*n* = 41)	*p*-Value
LVEDD (mm)	56.0 (51.0–63.0)	58.0 (52.5–26.5)	55.0 (50.0–63.0)	0.540
LVEF (%)	31.7 ± 9.6	28.7 ± 8.3	33.0 ± 9.9	0.108
LVEDV (mL)	206.0 (166.0–262.0)	194.0 (174.0–263.0)	129.0 (96.0–168.0)	0.697
LVESV (mL)	132.0 (104.0–177.0)	156.0 (126.0–178.0)	129.0 (96.0–168.0)	0.174
E/A	0.9 (0.6–1.7)	1.5 (0.7–2.0)	0.9 (0.6–1.4)	0.157
E/e′	11.5 (8.1–16.3)	12.1 (7.7–15.4)	11.1 (8.2–17.0)	0.905
E/E′ lateral	9.6 (6.5–13.8)	9.1 (5.8–12.5)	9.7 (6.7–16.0)	0.455
E/E′ septal	12.7 (8.73–17.4)	13.6 (9.1–18.1)	12.7 (8.8–17.3)	0.757
MV Dec T (ms)	171.0 (141.0–207.0)	150.0 (115.0–163.0)	195.0 (148.0–222.0)	0.006
S′ lateral (mm/s)	0.7 ± 0.2	0.5 ± 0.1	0.7 ± 0.2	0.005
S′ septal (mm/s)	0.6 (0.5–0.8)	0.5 (0.4–0.6)	0.7 (0.5–0.8)	0.072
LA area (cm^2^)	21.4 (18.0–26.0)	25.8 (24.2–27.9)	19.7 (17.3–25.1)	0.061
MAPSE (mm)	12.3 ± 2.8	11.5 ± 2.3	12.7 ± 2.9	0.106
Cardiac output (L/min)	4.9 ± 1.3	5.2 ± 1.5	4.8 ± 1.2	0.218

LVEDD—left ventricular end-diastolic diameter, LVEF—left ventricular ejection fraction, LVEDV—left ventricular end-diastolic volume, LVESV—left ventricular end-systolic volume, E/A—peak velocity of blood flow during left ventricular relaxation in early diastole/peak velocity of flow in late diastole caused by atrial contraction, E/e′—left ventricular transmitral early diastolic filling velocity/left ventricular early diastolic myocardial velocity, E/E′ lateral—peak velocity of blood flow during left ventricular relaxation in early diastole/lateral left ventricular early diastolic myocardial velocity, E/E′ septal—left ventricular transmitral early diastolic filling velocity/septal wall of left ventricular early diastolic myocardial velocity, MV Dec T—mitral valve deceleration time, S′ lateral—systolic excursion velocity of the lateral wall of the left ventricle, S′ septal—systolic excursion velocity of the septum of the left ventricle, LA area—left atrium area, MAPSE—mitral annular plane systolic excursion. Normally distributed data were presented as mean ± standard deviation and compared using the independent samples *t*-test. For non-normally distributed data, values were expressed as median (interquartile range) and compared using the Mann–Whitney U test. The threshold for statistical significance was set at *p* < 0.05.

**Table 2:** Patient characteristics and Holter ECG parameters in patients with and without diabetes.

Characteristic	All Sample (*n* = 60)	Patients with Diabetes (*n* = 19)	Patients without Diabetes (*n* = 41)	*p*-Value
NYHA class	I—4 (7%)	I—0 (0%)	I—4 (10%)	0.136
	II—33 (55%)	II—9 (47%)	II—24 (58%)	
	III—21 (35%)	III—10 (53%)	III—11 (27%)	
	IV—2 (3%)	IV—0 (0%)	IV—2 (5%)	
NTproBNP (pg/mL)	2568 (634–6193)	3598 (1767–8006)	1990 (553–5733)	0.211
LVP (%)	Yes—26 (43%)	Yes—6 (43%)	Yes—20 (43%)	0.211
	No—34 (57%)	No—13 (57%)	No—21 (57%)	
SDNN (ms)	76.0 (55.5–106.0)	68.0 (51.0–88.5)	80.0 (63.0–107.0)	0.252
SDANN (ms)	69.3 ± 29.8	69.1 ± 34.8	69.5 ± 27.6	0.961
SDNN index (ms)	34.0 (27.0–51.0)	29.0 (24.0–40.0)	38 (31.0–52.0)	0.171
RMSSD	25.0 (15.8–36.5)	28.0 (17.5–36.0)	22.0 (15.0–38.0)	0.395
PNN50 (%)	3.5 (0.0–10.0)	5.0 (1.0–9.0)	3.0 (0.0–10.0)	0.445
Triangular Index (ms)	17.4 (12.6–24.6)	14.9 (11.6–17.2)	19.6 (13.2–26.6)	0.035
vlF (Hz)	898 (592–1765)	678 (275–1926)	958 (702–1759)	0.194
lF (Hz)	181.0 (93.9–457.0)	121.0 (85.8–468.0)	193.0 (108.0–366.0)	0.581
hF (Hz)	91.4 (37.7–182.0)	104.0 (50.1–192.0)	65.9 (29.5–178.0)	0.394
Deceleration capacity (ms)	4.0 ± 1.9	2.9 ± 1.6	4.5 ± 1.8	0.002
Acceleration capacity (ms)	−4.0 (−5.6–−3.0)	−3.6 (−4.5–−3.0)	−4.3 (−5.8–−3.0)	0.132
TWA (%)	Yes—12 (20%)	Yes—5 (43%)	Yes—7 (43%)	0.405
	No—48 (80%)	No—14 (57%)	No—34 (57%)	

SDNN—standard deviation of RR intervals for the entire duration, SDANN—standard deviation of the averages of NN intervals in each 5 min segment across the entire recording, SDNN index—mean of the 5 min normal-to-normal intervals throughout the complete recording, RMSSD—square root of the mean of the squares of the successive differences between adjacent NN intervals, PNN50—ratio of NN50 to the total count of NN intervals, vlF—very low frequency, lF—low frequency, hF—high frequency, NTproBNP—amino-terminal pro-brain natriuretic peptide, LVP—late ventricular potentials, TWA—T wave alternans.

**Table 3:** The relationship between creatinine clearance, body mass index, uric acid, and Holter ECG Parameters.

Characteristic	Creatinine Clearance	Body Mass Index	Uric Acid
SDNN (ms)	Spearman’s ρ = −0.068 *p*-value = 0.605	Spearman’s ρ = 0.167 *p*-value = 0.203	Spearman’s ρ = −0.107 *p*-value = 0.416
SDANN (ms)	Pearson’s r = −0.103 *p*-value = 0.425	Pearson’s r = 0.143 *p*-value = 0.277	Pearson’s r = −0.063 *p*-value = 0.632
SDNN index (ms)	Spearman’s ρ = −0.014 *p*-value = 0.917	Spearman’s ρ = 0.038 *p*-value = 0.774	Spearman’s ρ = −0.102 *p*-value = 0.437
RMSSD	Spearman’s ρ = −0.287 *p*-value = 0.026	Spearman’s ρ = 0.065 *p*-value = 0.621	Spearman’s ρ = 0.029 *p*-value = 0.825
PNN50 (%)	Spearman’s ρ = −0.318 *p*-value = 0.013	Spearman’s ρ = 0.088 *p*-value = 0.505	Spearman’s ρ = 0.105 *p*-value = 0.423
Triangular index (ms)	Spearman’s ρ = 0.041 *p*-value = 0.758	Spearman’s ρ = 0.167 *p*-value = 0.203	Spearman’s ρ = −0.207 *p*-value = 0.112
vlF (Hz)	Spearman’s ρ = 0.019 *p*-value = 0.886	Spearman’s ρ = −0.066 *p*-value = 0.618	Spearman’s ρ = −0.032 *p*-value = 0.810
lF (Hz)	Spearman’s ρ = 0.08 *p*-value = 0.949	Spearman’s ρ = −0.031 *p*-value = 0.812	Spearman’s ρ = 0.003 *p*-value = 0.979
hF (Hz)	Spearman’s ρ = −0.287 *p*-value = 0.026	Spearman’s ρ = 0.020 *p*-value = 0.882	Spearman’s ρ = −0.011 *p*-value = 0.933
Deceleration capacity (ms)	Pearson’s r = 0.005 *p*-value = 0.967	Pearson’s r = −0.131 *p*-value = 0.317	Pearson’s r = −0.260 *p*-value = 0.045
Acceleration capacity (ms)	Spearman’s ρ = −0.037 *p*-value = 0.777	Spearman’s ρ = −0.038 *p*-value = 0.771	Spearman’s ρ = 0.229 *p*-value = 0.078

SDNN—standard deviation of RR intervals for the entire duration, SDANN—standard deviation of the averages of NN intervals in each 5 min segment across the entire recording, SDNN index—mean of the 5 min normal-to-normal intervals throughout the complete recording, RMSSD—square root of the mean of the squares of the successive differences between adjacent NN intervals, PNN50—ratio of NN50 to the total count of NN intervals, vlF—very low frequency, lF—low frequency, hF—high frequency.

**Table 4:** Comparison between patients with and without LVP.

Characteristic	LVP (*n* = 26)	No LVP (*n* = 34)	*p*-Value
Creatinine clearance (mL/min/1.73 m^2^)	78.5 (55.0–98.5)	91.5 (64.0–102.0)	0.412
Body mass index (kg/m^2^)	27.6 (25.1–30.1)	28.5 (25.4–31.4)	0.748
Uric acid (mg/dL)	6.7 (5.62–8.8)	6.0 (5.2–8.1)	0.165

LVP—late ventricular potentials.

**Table 5:** Comparison between patients with and without TWA.

Characteristic	TWA (*n* = 12)	No TWA (*n* = 48)	*p*-Value
Creatinine clearance (mL/min/1.73 m^2^)	87.0 (57.3–91.5)	85.0 (59.0–103.0)	0.427
Body mass index (kg/m^2^)	29.1 (25.9–32.1)	28.2 (25.1–30.8)	0.598
Uric acid (mg/dL)	6.2 (5.3–8.5)	6.3 (5.5–8.2)	0.978

TWA—T-wave alternans.

## Data Availability

The data presented in this study are available within the article.
